# Supplementary material for: scX: a user-friendly tool for scRNAseq exploration
Source: Bioinform Adv. 2024 May 2;4(1):vbae062. doi: 10.1093/bioadv/vbae062 (PMC11109472; doi:10.1093/bioadv/vbae062)
Supplement: vbae062_Supplementary_Data [file vbae062_supplementary_data.zip › scX_supMat.pdf]

scX: A user-friendly tool for scRNA-seq exploration

Supplementary Material

	scX	ShinyCell	cellxgene	iSEE	UCSC Cell Browser	SCHNAPPs	SEQUIN	ShIVA	SingleCellAnalyzer	CellSnake	ASAP	Loupe Browser
SC analysis	x					x	x	x	x	x	x	
SC analysis (interactive)						x	x	x	x		x	x
Web sharing	x	x	x	x	x	x	x	x	x		x	
Docker	x		x	x				x		x	x	
Cloud Support	x	x	x			x		x				
SaaS					x		x		x		x	x
Dataset Summary	x					x	x	x			x	x
QC plots	x					x	x	x	x			
Explore covariates (categorical)	x	x	x			x	x	x				
Explore covariates (continuous)	x	x	x	x		x	x					
Multiple embeddings	x	x	x	x	x	x	x	x	x		x	x
3D plots	x					x					x	x
Interactive plots	x		x	x	x	x		x	x		x	x
Side-by-side embeddings		x		x	x				x			
Differential Expression	x		x			x	x	x	x		x	x
on-the-fly cell-selection for marker identification	x		x	x		x	x				x	x
Gene Co-expression	x	x	x			x					x	x
Input of ad-hoc gene list	x											x
Multiple genes bubbleplots / heatmaps	x	x		x	x	x	x		x			x
Order heatmap by covariate (continuous)	x			x								
Order heatmap by covariate (categorical)	x			x		x	x		x			x
Zoom in/out	x		x	x	x	x		x			x	x
Export images PDF/PNG	x	x			x	x	x	x	x		x	x
SCE object	x	x		x		x						
Seurat object	x	x			x			x				x
txt/csv	x	x			x	x	x				x	
h5ad file		x	x		x							
loom file		x			x						x	
cellRanger	x							x		x		
Platform	R	R	Python	R	Python	R	R	R	Webpage	R/Python	R/Java/Python	App/R

Table ST1 Comparison between scRNAseq analysis tools based on functionalities (orange), implemented analyses and plot versatility (blue), and supported input file formats (green). Features were selected in the spirit of the comparison presented by Ouyang and collaborators [Ouyang2021]. ‘SC analysis’ refers to the capability of entering a raw object and performing a complete single-cell analysis (normalization, dimReduction, clustering, marker selection); ‘interactive’ refers to the capability of conducting the whole analysis within the app. ‘Web-sharing’ refers to the ability to host the data on a web page. ‘Docker’ indicates whether a Docker image is included with the tool. ‘Cloud support’ indicates whether instructions are available to deploy the application on a public cloud. ‘SaaS’ (Software as a Service) indicates whether the tool is hosted, allowing users to upload their data and interactively process it.

N	Size	Preprocess			Markers tab	Heatmap w/o cell clustering		Heatmap w/ cell clustering	
		scX size	Memory (GB)	Time	Memory (GB)	Memory (GB)	Approx time	Memory (GB)	Approx time
			2.76	0:03:45	0.49	0.47		2.92	
5000	126.5 MB	619.8 MB	3.00	0:04:07	0.49	0.46	0:00:04	2.80	0:00:23
			2.77	0:04:02	0.48	0.46		2.72	
			3.55	0:07:43	0.55	0.29		3.08	
10000	228 MB	894.2 MB	3.86	0:08:02	0.54	0.49	0:00:06	3.59	0:00:35
			3.33	0:08:02	0.54	0.49		3.59	
			4.86	0:17:43	0.79	0.68		5.34	
20000	431 MB	2.7 GB	4.86	0:17:45	0.86	0.68	0:00:05	5.89	0:01:15
			5.34	0:17:46	0.86	0.68		5.89	
			6.34	0:36:44	1.17	0.8		4.00	
40000	837.8 MB	4 GB	6.34	0:38:50	1.19	0.9	0:00:10	4.02	0:03:06
			6.26	0:38:56	1.19	0.8		4.00	
			11.70	1:33:35	2.11	1.03		12.09	
80000	1.9 GB	8.1 GB	12.02	1:25:52	2.04	1.07	0:00:16	12.05	0:07:16
			11.94	1:26:13	2.04	1.07		10.52	
			23.58	2:41:58	3.96				
160796	3.3 GB	12.4 GB	23.58	2:40:17	5.06	#N/A	#N/A	#N/A	#N/A
			23.59	2:44:54	5.06				
80000(*)	1.9 GB	8.1 GB			6.67	6.68	0:00:14	11.30	0:03:30
160796(*)	3.3 GB	12.4 GB			7.4	7.4	0:00:13	10.68	0:03:00

Table ST2 scX running times and peak memory consumption for sub-sampled datasets from single-cell RNA-seq data from Zeisel et al. (2018). Tests (by triplicates) were performed in an Intel(R) Xeon(R) Silver 4116 CPU @ 2.10GHz with 514G total RAM. Number of cells for each subset (N) are displayed in the first column. The in-memory size of the initial R object (Size) and the object created by the preprocessing function (scX size) are shown in the second and third columns. Peak memory usage in GB and running time for the pre-processing step are shown in the 4th and 5th columns. The last 5 columns report these quantities for 3 different tasks performed in the interactive Shiny platform (Markers tab does not include duration because it involves interactive use). Heatmaps for the two larger datasets could not be generated at single-cell levels. The 2 final rows correspond to a random subsampling of 50k cells for plots in the app (default in scX for large datasets).

Details:
Preprocessing: Data was preprocessed with 3 partitionVars arguments ("Age", "Class", "OriginalClusters") for DEGs and markers analysis, and 1 metadataVars ("MitoRiboRatio").

Tab Markers -> Cluster markers: 3D scatter plot colored by partition, marker list for 1 cluster, expression plots for 1 marker gene (3D scatter plot by expression, violin and spike plots)

Tab EDA -> Fields: Heatmap for all fields (nCounts, nFeatures, MitoRiboRatio) with Cluster Row, without Cluster Column, colored by category "OriginalClusters"

Tab EDA -> Fields: Heatmap for all fields (nCounts, nFeatures, MitoRiboRatio) with Cluster Row and Cluster Column, colored by category "OriginalClusters"

References:

Zeisel A et al. (2018). Molecular architecture of the mouse nervous system. Cell 174(4), 999-1014. https://doi.org/10.1016/j.

Risso D, Cole M (2023). scRNAseq: Collection of Public Single-Cell RNA-Seq Datasets. https://doi.org/10.18129/B9.

R package version 2.16.0, https://bioconductor.org/

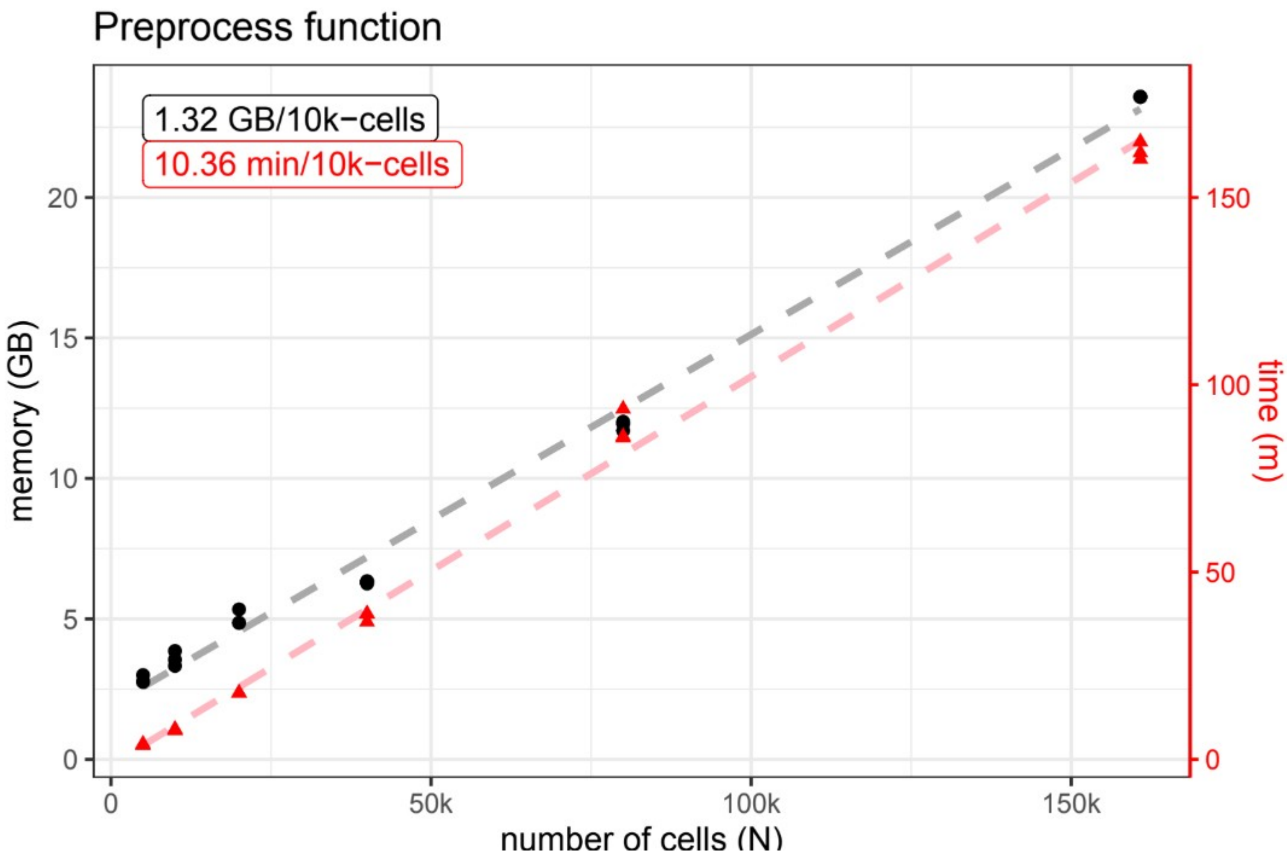
Preprocess function
1.32 GB/10k−cells
10.36 min/10k−cells
memory (GB)
0
5
10
15
20
time (m)
0
50
100
150
0
50k
100k
150k
number of cells (N)
